# N-Glycosylation and Inflammation; the Not-So-Sweet Relation

**DOI:** 10.3389/fimmu.2022.893365

**Published:** 2022-06-27

**Authors:** Barbara Radovani, Ivan Gudelj

**Affiliations:** ^1^ Department of Biotechnology, University of Rijeka, Rijeka, Croatia; ^2^ Genos Glycoscience Research Laboratory, Zagreb, Croatia

**Keywords:** N-glycosylation, N-glycans, inflammation, immunity, cytokines, leukocytes, immunoglobulins

## Abstract

Chronic inflammation is the main feature of many long-term inflammatory diseases such as autoimmune diseases, metabolic disorders, and cancer. There is a growing number of studies in which alterations of N-glycosylation have been observed in many pathophysiological conditions, yet studies of the underlying mechanisms that precede N-glycome changes are still sparse. Proinflammatory cytokines have been shown to alter the substrate synthesis pathways as well as the expression of glycosyltransferases required for the biosynthesis of N-glycans. The resulting N-glycosylation changes can further contribute to disease pathogenesis through modulation of various aspects of immune cell processes, including those relevant to pathogen recognition and fine-tuning the inflammatory response. This review summarizes our current knowledge of inflammation-induced N-glycosylation changes, with a particular focus on specific subsets of immune cells of innate and adaptive immunity and how these changes affect their effector functions, cell interactions, and signal transduction.

## Introduction

**Figure 1:**
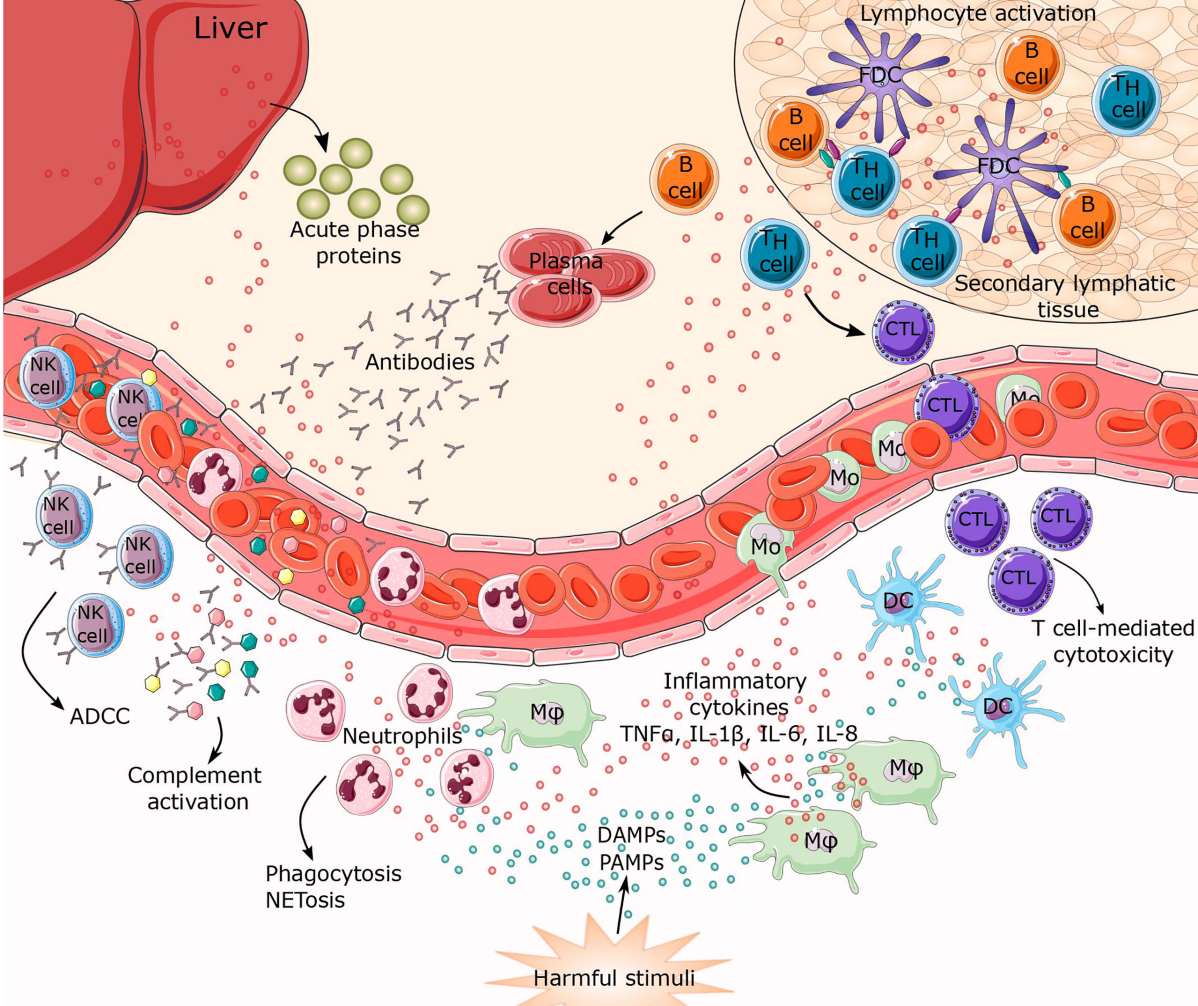
Inflammatory response to harmful stimuli. When tissue or cellular damage occurs, danger-associated molecular patterns (DAMPS), pathogen associated molecular patterns (PAMPs) and myriad inflammatory cytokines (TNFα, IL-1β, IL-6, IL-8) are released. These biomolecules can initiate activation of inflammatory pathways resulting in leukocyte recruitment of innate and adaptive immunity, thus establishing a highly coordinated network of many cell types. Activated macrophages, together with damaged endothelial cells, release factors that attract neutrophils and monocytes to the site of inflammation. This represents the first line of defense characterized mostly by phagocytosis and NETosis. Macrophages, together with mature dendritic cells (DCs), are specialized in exposing antigens to lymphocytes (T and B cells), thereby activating antigen-specific adaptive immunity. Lymphocyte differentiation leads to T cell-mediated cytotoxicity, antibody secretion, and antibody dependent cell cytotoxicity (ADCC). Simultaneously, cytokines trigger synthesis and secretion of acute phase proteins from the liver. CTL, cytotoxic T lymphocytes; FDC, follicular dendritic cells; Mφ, macrophage; Mo, monocyte; NK cell, natural killer cell.

**Figure 2:**
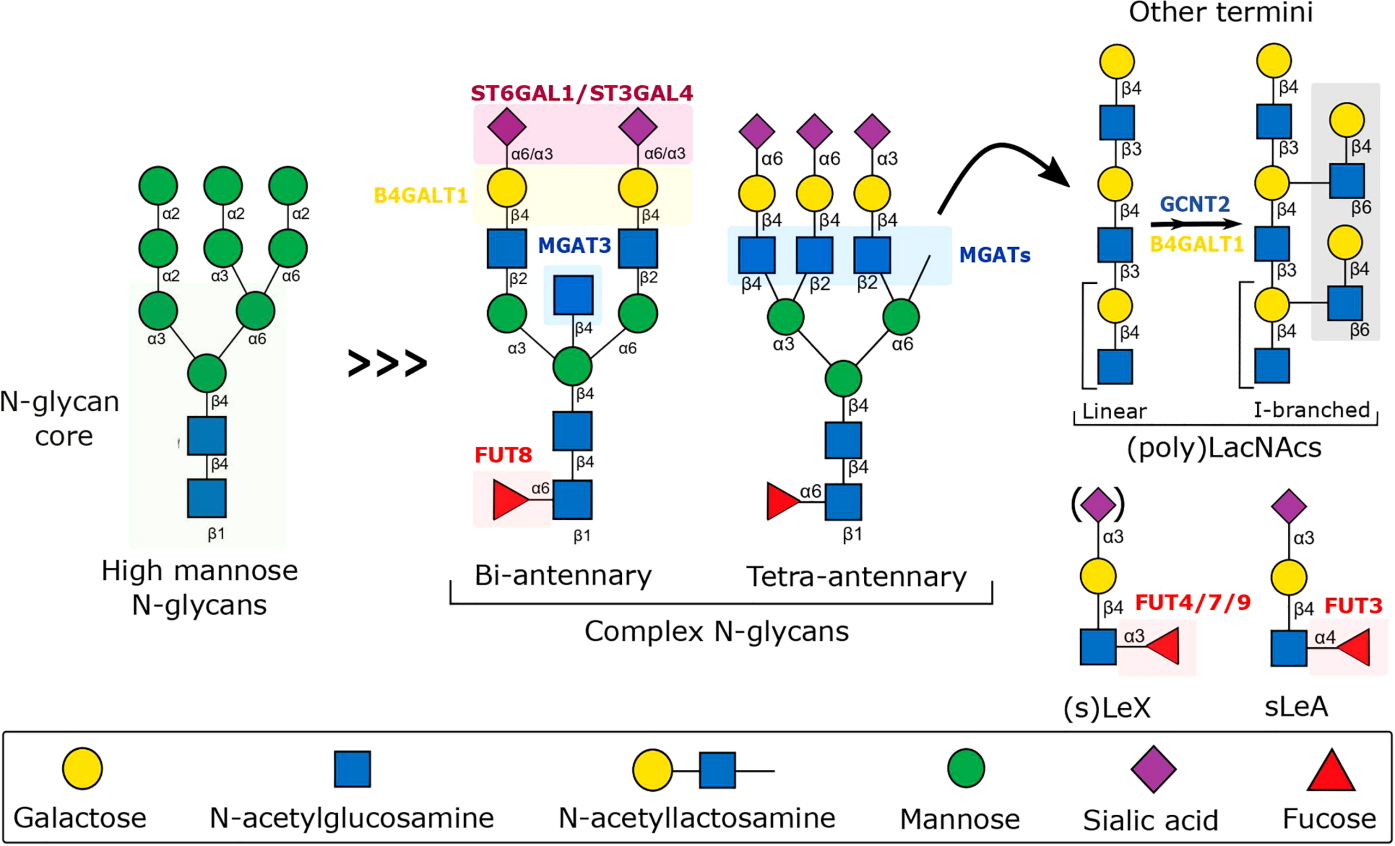
Schematic representation of the biosynthesis of N-glycans involved in the fine-tuning of the immune response to inflammation. The schematic includes the major N-glycan structures found on the surface of endothelium, immune cells, and secreted molecules, along with the relevant glycosyltransferases, whose expression has been shown to be modulated by inflammatory cytokines, dramatically affecting glycan-dependent interactions important for leukocyte immune regulation. B4GALT1, Beta-1,4-Galactosyltransferase 1; FUT, Fucosyltransferase; GCNT2, Glucosaminyl (N-acetyl) Transferase 2; MGAT, N-acetylglucosaminyltransferase; ST6GAL4, Beta-Galactoside Alpha-2,3-Sialyltransferase 4; ST6GAL1, Beta-Galactoside Alpha-2,6-Sialyltransferase 1.

Inflammation is part of a complex biological tissue response triggered by infectious, traumatic, toxic, or autoimmune injury. In acute inflammation, a controlled inflammatory response usually results in restoration of homeostasis. However, persistent induction and dysregulation of inflammation may contribute to the development of chronic inflammatory diseases (1). Chronic inflammation is characterized by numerous systemic physiological and biochemical changes, most of which are mediated by abundantly secreted proinflammatory cytokines (**Figure 1**). They are the key molecules responsible for triggering the proinflammatory potential of innate and adaptive immunity, oftentimes leading to tissue destruction (2). Moreover, chronic inflammation is characterized by marked changes in glycosylation (3, 4). Glycosylation is one of the most common posttranslational modifications of proteins and plays an important role in a variety of biological functions, including protein stability and effector functions, intercellular interactions, signal transduction, and cell immunogenicity. The enzymatic processes of protein glycosylation normally occur in the endoplasmic reticulum (ER) and Golgi apparatus, but can also occur in the cytoplasm and nucleus. The glycan structures are covalently linked to the protein backbone *via* the nitrogen atom of the asparagine or the oxygen atom of the serine/threonine side chains, forming N-linked and O-linked glycoproteins, respectively. The core of N-linked glycans consists of two consecutive N-acetylglucosamines (GlcNAc) and three mannoses, which can be further extended and modified by various glycosyltransferases (GTs) and glycosidases to form oligomannose, complex, or hybrid N-glycans (**Figure 2**) (5). N-glycans are found on the surface of key entities involved in the inflammatory response, including endothelial adhesion molecules, immune cells of innate and adaptive immunity, and secreted immunoglobulins and acute phase proteins (APP). The composition of their N-glycans has been shown to be modulated by abundantly secreted proinflammatory cytokines, presumably by regulating the expression of GTs and affecting the substrate availability required for N-glycan biosynthesis. Overall, the changes in N-glycosylation observed in chronic inflammation are diverse but strongly dependent on the particular subset of immune cells. Affected features of N-glycan structure include changes in the number of antennae, changes in N-glycan structure composition, and diversification of saccharide bonds resulting in different ligand epitopes. Consequently, altered N-glycosylation can significantly affect leukocyte trafficking, trigger a shift toward more proinflammatory effector functions of leukocytes, and initiate proinflammatory transformation of secreted immunoglobulins and APPs, ultimately leading to the development of various inflammatory diseases. Therefore, the aim of this review is to summarize what is known about the relationship between N-glycosylation and chronic inflammation, proinflammatory cytokines, and consequently the development of inflammatory diseases.

## Endothelium

One of the main functions of the endothelium is transportation of immune cells to the site of inflammation. To successfully pass through the endothelium, immune cells undergo complex process which involves ligand dependent binding followed by surface rolling, adhesion, and finally transendothelial migration (6, 7). Each step in this cascade is dependent on interaction between endothelial adhesion molecules and their counterreceptors expressed on the surface of leukocytes. Key players in leukocyte transmigration process are selections, integrins, intercellular and vascular adhesion molecules (ICAMs and VCAMs), platelet endothelial cell adhesion molecules (PECAMs), and junctional adhesion molecules (JAMs) (8). The majority of the endothelial adhesion molecules are heavily N-glycosylated (9), which is crucial for successful leukocyte trafficking, as defined by the “zip code” hypothesis. In the circulation, leukocytes encounter various proteins and sugars expressed on the endothelial surfaces. Efficient leukocyte adhesion is achieved only when a specific combination of an adhesion molecule protein and N-glycan is expressed (10). Adhesion molecules are not normally expressed in resting cells, however their expression is upregulated in inflammation, *via* cytokine-induced signaling pathway, such as NF-κB (11–14). Additionally, the life cycle of N-glycans involved in the leukocyte trafficking is significantly controlled by inflammation (15–17). Since inflammation-dependent modulation of adhesion molecule expression and N-glycome biosynthesis is critical for the innate immune response, dysregulation of this axis may be crucial for the transition from an innate immune response to inflammatory disease.

### Selectins

Selectins are calcium-dependent (C-type) lectins that recognize specific glycan residues as their ligands and mediate the adhesion of immune cells to the endothelium. To date, three members of the selectin family have been identified, P-selectin, E-selectin and L-selectin (18). L-selectin is highly expressed on hematopoietic stem cells and mature leukocytes, and is rapidly shed by proteolytic cleavage upon leukocyte activation (18–20). P- and E-selectin are known as “vascular selectins” because they can be expressed on endothelial cells. P-selectin is constitutively expressed by endothelial cells and platelets where they are stored in Weibel-Palade bodies and α-granules, respectively. Therefore, they can be translocated to the cell surface within minutes after a proinflammatory stimuli such as thrombin and histamine, making them the most important adhesive molecules in acute injury. On the other hand, E-selectin is not constitutively expressed by endothelial cells, but their expression is strongly upregulated by inflammatory cytokines such as interleukin 1β (IL-1β) and tumor necrosis factor α (TNFα) through binding of NF-κB to regulatory domains in the E-selectin promoter. The latter is not possible in the case of P-selectin, as the P-selectin promoter in humans lacks binding sites for NF-κB (21, 22). Because of this property, E-selectin is considered the most important adhesive molecule involved in leukocyte capture in chronic inflammation. The involvement of selectins in the development of many acute and chronic inflammatory conditions is dependent on the selectin-ligand axis, with N-glycosylation playing an important role. The interaction required for leukocyte capture on the endothelial surface is dependent on the affinity of selectins for sialofucosylated glycan epitopes expressed on both endothelial and immune cells. These include sialyl Lewis x (sLex), sialyl Lewis a (sLea), and 6-sulfo sialyl Lewis x (6-sulfo sLex) epitopes, which are responsible for mediating leukocyte capture/rolling during inflammation and are relevant to the successful homing of lymphocytes to lymph nodes (15, 23, 24). E-selectin binds to sialofucosylated N-glycans on E-selectin ligand-1 (ESL-1) and CD44 glycovariant, hematopoietic cell E-/L-selectin ligand (HCELL), to support leukocyte extravasation to the site of inflammation (25–27). Interestingly, Pachón-Peña et al. demonstrated the potential use of glycoengineered HCELL on human adipose-derived mesenchymal stem cells (A-hMSCs) to direct their migration to sites of tissue injury/inflammation, thus enabling relevant immunomodulatory and regenerative functions (28). In addition, the sLex epitope found on APPs may modulate the binding of leukocytes to E-selectin (29, 30). In the case of L-selectin, Mitoma et al. demonstrated a critical role for 6-sulfo sLex-decorated N-glycans found on CD34, a major component of the L-selectin ligand, in the leukocyte trafficking in the high-endothelial venules (HEV) of the peripheral lymph node (17). Interestingly, Huopaniemi et al. showed that co-regulated expression of CMP-sialic acid and GDP-fucose transporters, essential for the synthesis of 6-sulfo sLex, occurs in inflammation, which is not common in physiological conditions. Therefore, it has been suggested that there must be an inflammation-induced transcriptional regulation for Golgi membrane transporters that support trafficking of substrates necessary for synthesis of 6-sulfo sLex N-glycans (31). Furthermore, sulfonation of sLex epitopes on N-glycans is thought to be the result of N-acetylglucosamine-6-O-Sulfotransferase-1 (GlcNAc6ST-1) activity, but further studies are needed to uncover how this synthesis is regulated under physiological and pathological conditions (32). Moreover, Beta-Galactoside-Alpha-2,3-Sialyltransferase 4 (ST3GAL4) is the primary sialyltransferase regulating the synthesis of sLex epitopes in human myeloid leukocytes (33), the expression of which, together with the expression of 6-sulfo sLex, has been shown to be increased by TNFα in chronic lung disease. Thus, disruption of ST3GAL4 function in human myeloid cells may represent a potential target for anti-cell adhesion and anti-inflammatory therapy (16). In addition, fucosyltransferases such as FUT7 and FUT9 are involved in the synthesis of the Lex epitope, and FUT7^-^9^-^ dual knockdown has been demonstrated to significantly decrease the selectin-dependent interaction between leukocytes and endothelial cells (34). Interestingly, an *in vivo* study has shown that the cytokines IL-6 and/or IL-8 can induce a significant increase in the expression of alpha-1,3/4-fucosyltransferases in mucosal cells, contributing to an increased level of sLex epitopes and thus to dysregulated transendothelial migration. The latter might potentiate the persistent lung inflammation and tissue damage in cystic fibrosis (CF) (35).

### ICAM-1 and VCAM-1

After leukocyte rolling and capture by selectins, firm endothelial adhesion of leukocytes is mediated by ICAM-1 and VCAM-1. They are members of the immunoglobulin supergene family that are expressed during chronic inflammation on vascular endothelium, lymphocytes, and macrophages (36). The expression of ICAM-1 and VCAM-1 is under the direct influence of proinflammatory cytokines, such as TNFα, which increase their levels on the endothelial surface (37). In response to inflammation, increased ICAM-1 and VCAM-1 cell surface levels enhance adhesive interactions with their ligands on leukocytes, Lymphocyte function-associated antigen 1/Macrophage-1 antigen (LFA-1/MAC-1) and Very late antigen-1 (VLA-1), respectively (38, 39). N-glycosylation is a crucial factor that can significantly affect the ligand binding of ICAM-1 and VCAM-1 (38, 40–43). In general, the transition from the homeostatic to the inflammatory state of the endothelium is characterized by a decrease in N-glycan complexity and increased expression of high mannose and hybrid structures (44–47). This has been demonstrated to be a consequence of proinflammatory stimulation, possibly by inhibition of early mannose-trimming enzymes (α-mannosidase) (42). Not surprisingly, increased presence of high-mannose ICAM-1 (HM-ICAM-1) results in high-affinity leukocyte binding (38). In particular, this phenomenon is seen in CD16^+^ proinflammatory monocytes, which have a higher affinity for HM-ICAM-1 molecules in atherosclerotic lesions compared with complex α-2,6-sialylated ICAM-1 (48, 49). However, this is not the only mechanism by which the pathological state is maintained in chronic inflammation, as far as N-glycosylation is concerned. In systemic lupus erythematosus (SLE), diabetes, and rheumatoid arthritis (RA), this is regulated by inhibition of galectin-mediated immunosuppressive prevention of ICAM-1/LFA-1 interaction (50), aberrant expression of ICAM-1 N-glycans due to high glucose (51), and activity of glycosyltransferases (52). Another possible candidate for inflammatory modulation is VCAM-1, where removal of α2,6-sialic acid increases leukocyte trafficking (41). The mechanism of action does not involve stronger binding with VLA-1 on leukocytes but with galectin-3 (Gal-3), which supports leukocyte adhesion (53).

### PECAMs

The final step in the process of leukocyte extravasation is transendothelial migration (TEM). Adhesion molecules mainly associated with this phenomenon are PECAMs (54). PECAM-1 is a member of the immunoglobulin (Ig) superfamily selectively expressed on the surface of immune cells and is highly enriched at the intercellular interface of endothelial cells (54). The main mechanism responsible for interaction of PECAMs with leukocytes involves homophilic binding (55). As PECAM-1 is highly N-glycosylated, Lertkiatmongkol and her group showed that homophilic binding interactions of human PECAM-1 are supported by α2,3-linked, but inhibited by α2,6-linked sialic acid residues (56). This was previously demonstrated by Doring et al. who presented solid evidence for the importance of α2,3-linked sialic acid in leukocyte activation, adhesion, and recruitment to inflamed vessels (57). In agreement with this, it was later shown that a variety of proinflammatory cytokines secreted in chronic inflammation can downregulate the levels of the (extracellular) enzyme Beta-Galactoside Alpha-2,6-Sialyltransferase 1 (ST6GAL1), responsible for the synthesis of α2,6-linked sialic acid, and by that way maintain the inflammatory state (58, 59).

## Innate Immunity

Innate immunity is the first line of defense against infection and consists of resistance mechanisms that are not specific to any pathogen. In any infection or tissue injury, inflammation is triggered when innate immune cells recognize molecular patterns that are foreign to a tissue, called pathogen-associated molecular patterns (PAMPs), and initiate a cascade of inflammatory responses. These innate immune cells include tissue-derived macrophages, natural killer cells (NK cells) and dendritic cells (DCs), as well as circulating leukocytes such as monocytes and neutrophils (60, 61). To communicate with other immune cells and exert their immunomodulatory functions, they often rely on N-glycans expressed on their surface and counterreceptors expressed by their binding partners. In this section, we present examples of altered N-glycosylation in innate immune cells due to chronic inflammation and show how this is reflected in the functionality of immune cells and consequently in the progression of various chronic inflammatory conditions.

### Neutrophils

**Figure 3:**
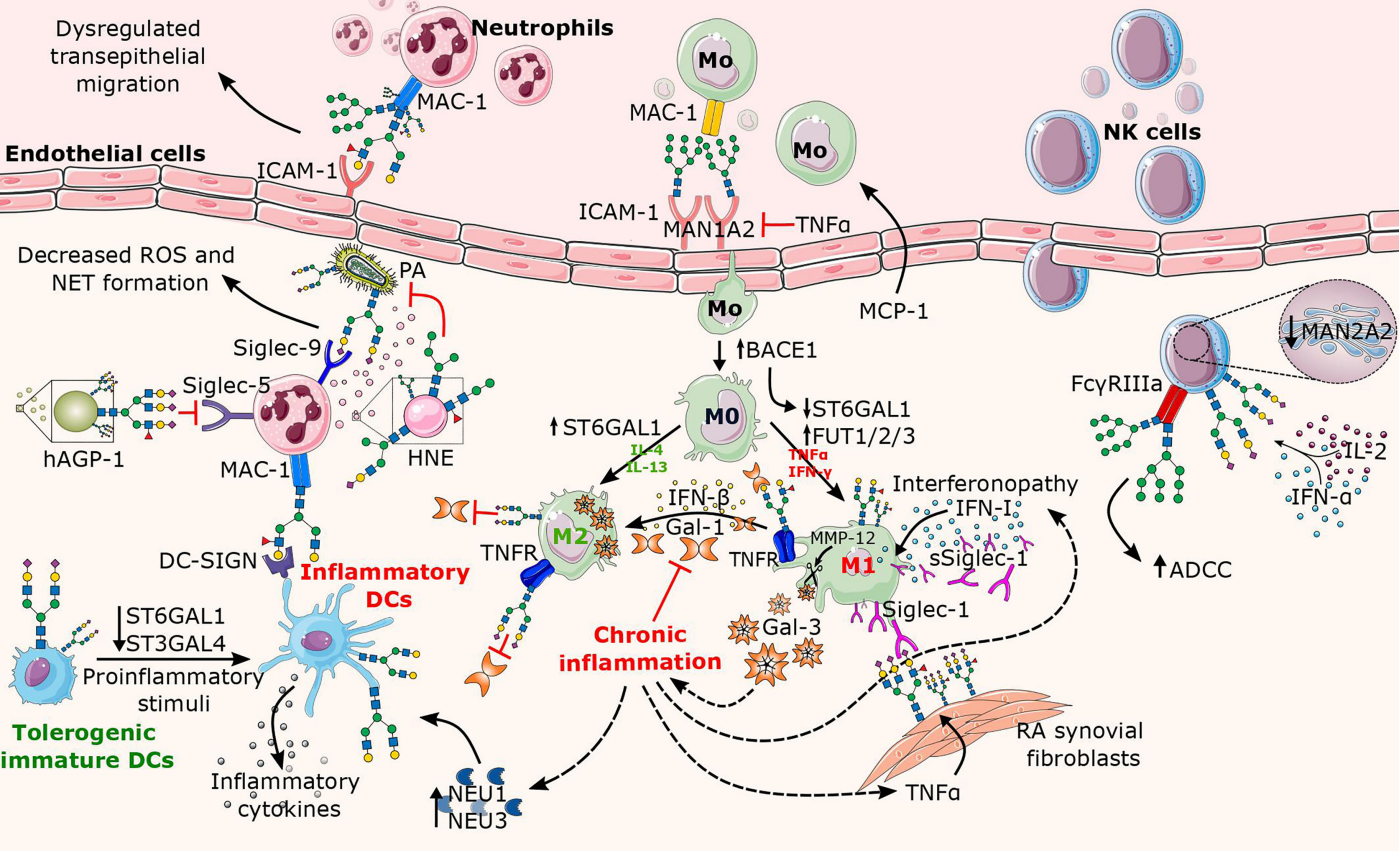
Overview of altered N-glycosylation pathways in innate immune cells during chronic inflammation. The major factors contributing to the alterations in N-glycosylation are proinflammatory cytokines (e.g., TNFα, IL-2, IFN-α, IFN-γ) that are released in excess during inflammation. Here, the affected structural elements of N-glycans on the surface of innate leukocytes (neutrophils, macrophages, NK cells, and DCs) are shown along with their associated glycosyltransferases and glycosidases. In neutrophils, the increase of the Lex motif on the integrin MAC-1 leads to dysregulated neutrophil migration, whereas the binding of Lex decorated MAC-1 to DC-SIGN further triggers the activation of DCs. While neutrophilic granules (e.g., HNE) secreted by neutrophils carry truncated N-glycans, the presence of sialylated complex N-glycans and/or the sLex motif on Siglec counterbinding entities contributes to the inflammatory potential of neutrophils in a context-dependent manner. Proinflammatory cytokines enhance transport of monocytes and direct their differentiation into proinflammatory M1 macrophages, while contributing to the absence of sialylated N-glycans, cleavage of Gal-3, and increase in Siglec-1 expression. While surface-bound Siglec-1 is involved in the autoimmune response in rheumatoid arthritis (RA), soluble Siglec-1 is a marker in interferonopathy. In addition, the Gal-1/IFN-β feedback loop involved in termination of inflammation appears to be dysregulated in chronic inflammation. Similarly to macrophages, mature DCs also lack terminal sialic acids, plausibly due to inflammation-mediated decrease in sialyltransferase and/or increase in neuraminidase activity. As for NK cells, the presence of oligomannose N-glycans on FcγRIIIa significantly increases ADCC, whereas cytokine-induced increase in sialylation abrogates Siglec-9-dependent NK cell inhibition by cis-binding. BACE1, Beta-Site APP-Cleaving Enzyme 1; Gal, galectin; hAGP-1; hepatic α1-acid glycoprotein; HNE, human neutrophilic elastase; IFN, interferon; IL, interleukin; ICAM-1, intercellular adhesion molecule 1; MAC-1, macrophage-1 antigen; Man, Mannosidase; MMP-12, matrix metalloproteinase 12; MCP-1, monocyte chemoattractant protein-1; NEU, neuraminidase; NAGP-1, neutrophil α1-acid glycoprotein; PA, *Pseudomonas aeruginosa*.

Neutrophils are polymorphonuclear leukocytes that have long been known to be key players in pathogen recognition and elimination in acute inflammation, but their role in chronic inflammatory and autoimmune diseases, such as psoriasis, RA and SLE, has also been described (62, 63). Understanding the underlying mechanisms of neutrophil activation, migration, survival, and executive function may open new avenues for the treatment of chronic inflammation. N-glycosylation has been shown to contribute to important effector functions of neutrophils, such as extravasation, phagocytosis, degranulation, and formation of neutrophil extracellular traps (NETs) (64) (**Figure 3**). On their surface, neutrophils express the N-glycosylated MAC-1 integrin, which consists of two subunits - CD11b and CD18. This complex is involved in the regulation of neutrophil trafficking and interaction with other immune cells (65). Kelm et al. performed an analysis of glycan epitopes expressed on the neutrophil MAC-1 surface and observed decreased sialylation together with an increase in the Lex motif and high mannose content in chronic inflammation. These changes were significant in inflammatory bowel disease (IBD) because blocking the terminal Lex motif reduces dysregulated transepithelial migration of neutrophils, presumably by inhibiting the binding of MAC-1 to ICAM-1 molecules expressed on the surface of the inflamed epithelium (66, 67). Moreover, the Lex motif expressed on MAC-1 mediates binding to DC-SIGN expressed on DCs, thus providing an indirect link between innate and adaptive immunity. Monocytes and macrophages also express MAC-1, but since they lack the Lex motif, this trait is exclusively dependent on neutrophils (68). Therefore, the Lex motif may represent a novel target for modulating inflammation in chronic diseases in which tissue damage is mediated by dysregulated neutrophil trafficking. After neutrophils accumulate at the site of inflammation, their immunomodulation depends on efficient degranulation, phagocytosis, and NET formation. Interestingly, granule glycoproteins show differential, stage-dependent glycosylation that decorates them with hyper-truncated chitobiose core, paucimannose and complex monoantennary N-glycans (64). Interestingly, it was recently demonstrated that N-acetyl-Beta-D-Hexosaminidase (Hex) enzyme is elevated in many inflammatory diseases (69–71) and catalyzes formation of paucimannosidic glycans found on neutrophilic granules (72). Those paucimannosidic N-glycoforms of human neutrophil elastase (HNE) show an enhanced ability to suppress the growth of *P. aeruginosa* (PA), presumably by bypassing interactions with its suppressive counter-binding partner, A1-antitripsyne (A1AT) (73). However, PA was demonstrated to uptake host’s sialylated N-glycans, making these bacteria suitable binding partners for the inhibitory siglec-9 receptor expressed on neutrophils. As a result, neutrophils show reduced levels of reactive oxygen species (ROS) and released elastases which leads to reduced formation of NETs and increases survival of PA (74), eventually leading to chronic lung inflammation and tissue destruction as seen in CF patients susceptible to respiratory infections caused by PA. Therefore, further studies are needed to draw conclusions about neutrophil glycosylation role in CF pathogenesis and completely illuminate these processes. Another modulatory potential of neutrophil activity lies in alpha-1-acid glycoprotein (AGP-1). AGP-1 can stimulate neutrophil activation by inducing an increase in cytosolic calcium concentration through interactions with the neutrophil receptors siglec-5 and/or siglec-14, which preferentially bind α2-3 or α2-6 sialic acid. The latter is presumably true for acute inflammation as hyperfucosylation of AGP-1 in chronic inflammation leads to increased expression of sLex on AGP-1, a motif that is not a ligand for siglec-5 nor siglec-14 (75). Furthermore, in addition to the liver, AGP-1 is expressed by activated neutrophils. In contrast to the N-glycans expressed on hepatic AGP-1 (hAGP-1), neutrophil-expressed AGP-1 (nAGP-1) carries mainly high-mannose, nonsialylated, and mono-sialylated N-glycans (76). Taken together, these data may indicate that chronic inflammation in some cases attenuates neutrophil recruitment and activation in favor of other, more potent leukocytes.

### Monocytes and Macrophages

Monocytes and tissue macrophages are part of the mononuclear phagocyte system, which plays a central role in inflammation through antigen presentation, phagocytosis, and cytokine-mediated immune modulation (77). These mononuclear leukocytes are considered hallmarks of the transition from acute to chronic inflammation, as their accumulation is the result of cytokine-induced neutrophil apoptosis and increased production of monocyte chemoattractant protein (MCP-1) (78). Not surprisingly, several studies have uncovered different mechanisms in which monocytes and macrophages are involved in the modulation and maintenance of chronic inflammation. In particular, these have been demonstrated in cardiovascular disease (CVD) (79), RA (80, 81), chronic obstructive pulmonary disease (COPD) (82), diabetes (83, 84), and IBD (85). In the last decade, N-glycosylation has gained much attention as a tool by which inflammation orchestrates the immune response of monocytes and macrophages. There are three main steps involved in the accumulation of macrophages in the inflamed environment: recruitment of monocytes from the circulation, differentiation into macrophages, and activation of macrophages at the site of inflammation (77). All three steps are under the direct influence of altered N-glycosylation (**Figure 3**). First, after monocytes are recruited by MCP-1 (83), their passage through the endothelial layer requires a complex system of interactions with heavily N-glycosylated adhesion molecules. Previous studies have shown that proinflammatory cytokines such as TNFα can upregulate the expression of adhesion molecules (ICAM-1, VCAM-1, E-selectin) (12, 13) and regulate their N-glycosylation (86). This was confirmed by Chacko et al., who further identified the mannosidases MAN1A2 and MAN1C1 as subjects of decreased expression by TNFα. These mannosidases catalyze the early removal of mannose, which is required for the conversion of high mannose to hybrid and subsequently complex N-glycans. Using THP-1 monocytes and PPARy ligands, they also demonstrated that the dual function of TNFα, stimulation of adhesion molecules and regulation of their N-glycosylation, is controlled by independent pathways, underscoring the importance of high-mannose N-glycans for monocyte trafficking (87). Recently, Regal-McDonald and his team specified that MAC-1 receptors expressed on the intermediate, proinflammatory, subclass of monocytes (CD14^+^CD16^+^) have a higher affinity for HM-ICAM-1 compared with the classical subclass (CD14^+^CD16^-^). They also showed that the monocytes bind with higher affinity to HM-ICAM-1 than to α2,6-sialylated ICAM-1 (48, 49). After migration through the endothelium, monocytes differentiate into M0 macrophages, which can further polarize into different subclasses of macrophages stimulated by different cytokines - classically activated M1 macrophages, alternatively activated M2 macrophages, CD169 macrophages, or TCR macrophages (88). During differentiation from monocytes to macrophages, ST6GAL1 is downregulated, resulting in the loss of α2,6-sialic acid. Importantly, ST6GAL1 downregulation results from cleavage by Beta-Site APP-Cleaving Enzyme 1 (BACE1), which is dramatically upregulated during macrophage differentiation (89). This may also occur during differentiation into M1 macrophages, as it has already been shown that M2 macrophages, associated with anti-inflammatory effects, exhibit higher ST6GAL1 production compared with M1 (90). Moreover, α2,6-sialylation is included in the regulation of macrophage survival. Liu et al. showed that ST6GAL1 mediated α2,6-sialylation of TNFα death receptor 1 (TNFR1) expressed on primary macrophages inhibits apoptosis (91). This discovery further highlights the protective properties of α2,6-sialylation due to its promotion of proinflammatory M1 apoptosis, and survival of anti-inflammatory M2. In addition to sialylation, fucosylation also affects M1/M2 polarization in chronic inflammation. In RA, M1 macrophages were shown to express 5-10 times more fucosyltransferases (FUTs), catalyzing terminal and subterminal fucosylation (FUT1, FUT3, FUT7, and FUT9), than their monocyte progenitors, whereas this was not observed for FUT8, responsible for core fucosylation (92).Interestingly, terminal fucosylation is important for the synthesis of the sLex epitope, which is considered proinflammatory (93), whereas core fucosylation has more anti-inflammatory properties (94). Inhibition of terminal FUTs such as FUT1/2 leads to a shift in M1 differentiation toward M2 macrophages (92). This study was the first to highlight the potential of terminal fucosylation as a novel hallmark of inflammatory M1 macrophages. Another subclass of macrophages worth mentioning is CD169^+^. These macrophages express high levels of CD169, also known as sialoadhesin or Siglec-1, and are strategically positioned at the entry site of lymphoid tissue, where they bind and remove pathogens from the lymphatic fluid and blood (95). In addition to “gatekeeper” CD169^+^ macrophages that constitutively express Siglec-1, its expression can be upregulated in other tissue macrophages upon exposure to a type I interferon (IFN-I) (96). Siglec-1 belongs to the sialic acid-binding immunoglobulin-like lectins (Siglecs) (97), preferentially binding to α2,3-linked sialic acids (98), and differing from other siglecs as it has a long extracellular region (17 Ig domains) that lacks intracellular signaling motifs (99). In their review, O’Neill et al. introduced Siglec-1 as a macrophage-specific marker of chronic inflammation and emphasized its contribution to cell-cell and cell-matrix interactions of macrophages in inflammation (100). Also, soluble Siglec-1 (sSIGLEC-1) has been presented as a marker of monocyte and macrophage activation as well as a marker of interferonopathy in SLE and other inflammatory disease (101). Recently, Wang et al. showed that there is a TNFα mediated reduction in α2,6-, but not α2,3-terminal sialylation in RA (59). The latter would support interactions of proinflammatory synovial fibroblasts with pathogenic macrophages *via* Siglec-1, whose expression is upregulated in macrophages in RA (59, 102). On the other hand, Tanno et al. showed decreased expression of Siglec-1 on alveolar macrophages in COPD, significantly reducing their phagocytic capabilities against microbial pathogens and thus maintaining the inflammatory state (103). This clearly demonstrates how inflammation alters glycosylation and glycan binding molecules to maintain inflammatory environment, rather than the other way around. Mesenchymal stem cells (MSCs) have recently gained much attention as potential macrophage immunomodulators in chronic inflammation. As mentioned previously, they can be α(1,3)-exofucosylated to express the sLex-decorated CD44 ligand (known as HCELL), allowing them to migrate to the site of inflammation (28, 104). Further evidence suggests that targets of MSC mediated immunosuppression include macrophages, as there is evidence of the ability of adipose derived MSCs to shift macrophages from the M1 to the M2 phenotype (105, 106). Whether binding of HCELL ligand to Siglec-1 expressed on macrophages may be a possible mechanism of immunosuppression, thereby stopping macrophage interaction with other immune cells, is a question that remains to be answered. Another family of glycan-binding proteins involved in macrophage-mediated immunomodulation are the galectins. Among the 15 galectins identified to date expressed by immune cells, galectin-1 (Gal-1) and galectin-3 (Gal-3) show significant expression in macrophages and monocytes (107, 108). Both Gal-1 and Gal-3 possess a conserved carbohydrate recognition domain (CRD) that recognizes glycans containing a terminal N-acetyllactosamine (Galβ1,4GlcNAc or LacNAc). However, the terminal α2,6-sialylation present on LacNAc prevents the binding of Gal-1, whereas this was not observed for Gal-3 (109). Since the contribution of Gal-1 and Gal-3 to immunomodulation has been discussed in great detail elsewhere (108, 110, 111), we were focused here only on recent discoveries regarding the interplay between these galectins and macrophages. According to several different studies, soluble Gal-3 is mainly associated with proinflammatory functions (112–114). However, Di Gregoli et al. in their recent work proposed a new mechanism in which high levels of circulating Gal-3 in inflammation could be a consequence of matrix metalloproteinase 12 (MMP-12) dependent cleavage of Gal-3 from the surface of macrophages. Moreover, Gal-3 negative macrophages tend to exhibit proinflammatory properties, whereas Gal-3 positive macrophages exhibit pro-resolving and profibrotic properties (115). The latter is consistent with previously published data that revealed a novel mechanism of alternative M2 macrophage activation through binding of Gal-3 to its heavily N-glycosylated CD98 membrane receptor while emphasizing the association between M2 macrophages and increased fibrosis (116). Thus, although Gal-3 does not show direct proinflammatory effects, it is still part of the axis in maintaining the inflammatory state. On the other hand, Gal-1 seems to be a “jack of all trades” in resolving inflammation (111). Regarding the macrophages, Gal-1 is known to promote the differentiation of macrophages into the M2 profile (111, 117, 118). Yaseen et al. recently uncovered a positive feedback loop involving interferon β (IFN-β) mediated expression of Gal-1 in proinflammatory macrophages, thereby promoting their reprogramming into a pro-resolving phenotype with high expression of IFN-β (119). However, the specific mechanism is still unknown. One possibility is autocrine stimulation of proinflammatory macrophages by binding self-expressed Gal-1 to N-glycan ligands on their surface that normally lack the inhibitory α2,6-linked sialic acid. The Gal-1/IFN-β feedback loop is thought to occur at the time of termination of acute inflammation, so any misstep (e.g., insufficient Gal-1 expression) could lead to the development of a chronic inflammatory state, as decreased Gal-1 has been observed in several chronic inflammatory states (120–122).

### Dendritic Cells

DCs are antigen-presenting cells with the ability to take up antigens in the periphery and expose them to lymphocytes, thus bridging the gap between innate and adaptive immune responses (123). A specific subset of DCs derived from monocytes (Mo-DC) plays a key role in inflammation (124). The surface of Mo-DCs is covered with glycoproteins decorated predominantly with sialylated glycans (125). Sialylation of DCs is regulated during both differentiation and maturation, and has been found to significantly affect DC functions such as antigen uptake, phagocytosis, and T cell priming (126) (**Figure 3**). Immature Mo-DCs are often tolerogenic because they have high levels of α2,6- and α2,3-sialylated N-glycans that are recognized by inhibitory siglecs (127) and galectins (128), respectively. By binding to α2,3-sialic acid-decorated CD43/CD45 clusters expressed on DCs, Gal-1 has been shown to support differentiation of tolerogenic DCs, thereby promoting interleukin 10 (IL-10) mediated T cell tolerance and suppression of autoimmunity (129). However, DC maturation in the presence of proinflammatory stimuli results in significant downregulation of expression and activity of ST6GAL1 and ST3GAL4 (130, 131), which may cause phenotype switch to inflammatory DCs. In contrast to the strictly tolerogenic activity of Gal-1, there are conflicting data regarding the control of inflammatory and tolerogenic DC phenotypes mediated by Gal-3 (128, 132, 133), reflecting the fact that this is a context- and tissue-dependent phenomenon. Additionally, by regulating T cell differentiation, DCs may also indirectly contribute to altered glycosylation of IgG molecules. Gringhuis et al. identified the molecular mechanism by which fucose specific triggering of DC-SIGN leads to increased Interleukin 27 (IL-27) expression by DCs, which promotes differentiation of T follicular helper (Tfh) cells known to affect IgG production by B cells (134, 135). Interestingly, DC-SIGN preferentially binds N-glycans with fucose incorporated into the Lex epitope (136), which is abundantly expressed by various immune cells during inflammation, suggesting another potential mechanism for maintaining the inflammatory state. While the presence of sialic acids has a tolerogenic effect on DCs, fully desialylated DCs exhibit a much more potent phenotype – high expression of major histocompatibility complex (MHC) molecules, secretion of inflammatory cytokines, phagocytosis, and activation of inflammatory T cells (137). Although the exact mechanism is still unclear, sialidases such as neuraminidase 1 and 3 (NEU1 and NEU3) are thought to contribute to the desialylation of DCs (138–140). This hypothesis is also supported by the fact that sialidases are abundant and involved in the pathology of many inflammatory diseases (141). However, Lübbers et al. have recently demonstrated an alternative pathway for the induction of tolerance by DCs independent of their sialylation status, driven by the immunoregulatory sialic acid-siglec axis. Specifically, binding of α2-3-sialic acid to Siglec-9 expressed on the surface of DCs alters metabolic pathways and cytokine signaling and reprograms DCs to enhance regulatory T cell/T helper type 1 (Treg : Th1) ratio balance (142). Collectively, these data highlight the importance of glycan recognition by DCs in controlling both inflammation and its resolution.

### Natural Killer (NK) Cells

NK cells are known for their role in cell-mediated cytotoxicity and secretion of proinflammatory cytokines (143), which are critical for both the promotion of inflammation and immune regulation (144). The effector functions of NK cells are regulated by a series of activating and inhibitory receptors expressed on their surface, with glycosylation playing a key role in receptor-ligand recognition (**Figure 3**). FcγRIIIa (CD16a) is the most abundantly expressed activating receptor on circulating NK cells (145), and its role in antibody dependent cell mediated cytotoxicity (ADCC) is well established (146). While it is established that modulation of IgG N-glycome significantly affects its binding to FcγRIIIa (94), Several studies made observations that underscore the importance of N-glycosylation of FcγRIIIa for IgG binding affinity. Tremendous increase in binding affinity of proinflammatory afucosylated IgG was observed when oligomannose N-glycans were present on FcγRIIIa (147, 148), which correlated with decreased expression of α-mannosidase in NK cells (149). Furthermore, higher levels of sialylated complex N-glycans on FcγRIIIa were shown to correlate with lower affinity for antibody binding (150), which was also observed for the activating NK cell receptor 2B4 (CD244) (151). In their recent review, Rosenstock and Kaufmann describe an important contribution of sialic acids to the functions of NK cells, both through the expression of sialic acid-binding receptors and by having sialic acids on their surface (152). Two of these receptors are Siglec-7 and Siglec-9, which have an inhibitory function on NK cells. While Siglec-7 mainly recognizes tumor-expressing gangliosides (153), Siglec-9 has a high affinity for α2,6- and α2,3-linked sialic acids, including the sLex epitope (154). Cytokines such as interleukin 2 (IL-2) and interferon α (IFN-α) have been shown to increase the level of sialylation on the surface of NK cells (155, 156). Although increased sialylation is usually considered to be anti-inflammatory, the functional role of these sialic acids may be to mask Siglec-9 through *cis* interactions, and thus preventing the inhibition of NK cells that would occur through *trans* binding of sialic acids. The importance of Siglec-9 in NK cell immunoregulation has been demonstrated in liver inflammation, where decreased Siglec-9 expression has been associated with disease progression (157). Although glycosylation in NK cells is functionally important, there is little information on the underlying mechanisms that alter N-glycosylation of NK cells during inflammation because of their relatively low abundance. However, the development of methods to enrich human NK cells from a single donor (149) may be a first step toward a more detailed analysis of inflammation induced N-glycosylation changes in NK cells.

## Adaptive Immunity

In contrast to innate immunity, adaptive immunity is characterized by high degree of specificity as well as the substantial property of memory. The adaptive immune system can be further divided into cellular immunity mediated by T cells and humoral immunity represented by B cells and secreted antibodies (158). In adaptive immunity, glycans are essential for the majority of signal transduction and cell-cell interactions. N-glycans have been shown to regulate important steps in lymphocyte biology, such as T and B cell activity and cell differentiation and proliferation. Moreover, N-glycans are of great importance for the fate and function of secreted antibodies in chronic inflammation. In this section, we will therefore describe mechanisms by which inflammation can alter N-glycosylation of lymphocytes and antibodies, explain the significance of these changes in chronic inflammatory diseases, and discuss the potential of immunotherapies based on manipulation of the altered N-glycosylation.

### T Cells

**Figure 4:**
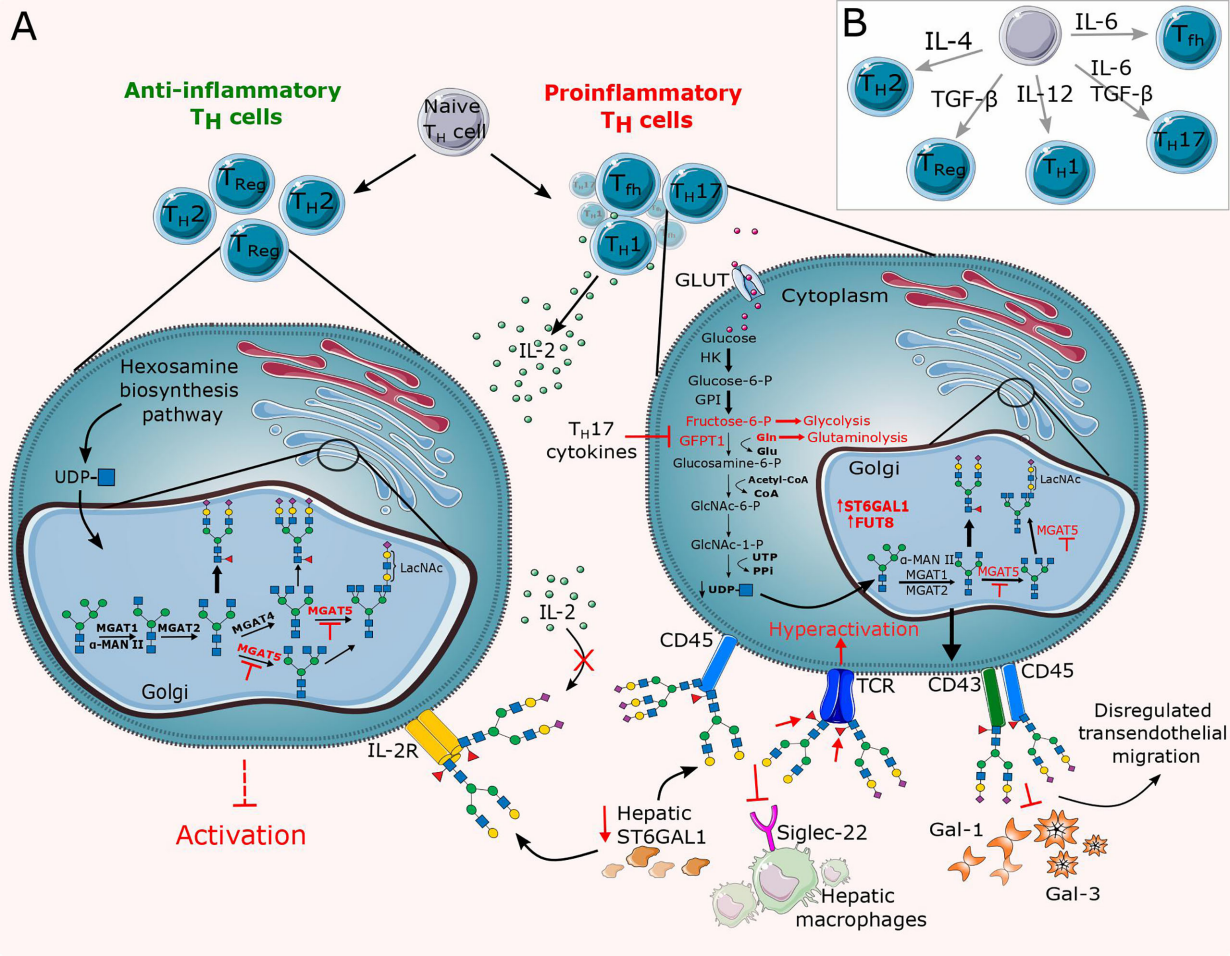
Overview of altered N-glycosylation pathways regarding T cells during chronic inflammation. **(A)** Differentiation of lymphocytes and thus their surface N-glycome is under the direct influence of cytokines and stimulation by antigen presenting cells (APCs). Cytokines control differentiation in favor of proinflammatory T cells (Th1, Th17, Tfh), thereby altering their N-glycome by dysregulating the expression of glycosyltransferases such as MGAT5, ST6GAL1 and FUT8 and abrogating substrate availability for the hexosamine biosynthesis pathway (HBP). The resulting N-glycan changes significantly reduce the binding affinity of inhibitory galectins and Siglecs. **(B)** Schematic representation of the relevant cytokines responsible for the T cell differentiation. GLUT, glucose transporter; TCR, T cell receptor; Tfh, T follicular helper cell; Th, T helper cell; Treg, T regulatory cell.

T cells (T lymphocytes) have a central role in the adaptive immune system. Briefly, after differentiation from thymocytes to naïve T cells, T cells leave the thymus and enter the periphery. There, exposure to antigens by antigen presenting cells (APCs) such as macrophages and/or DCs along with concomitant cytokine stimulation triggers maturation of naïve T cells. In general, mature T cells carry a unique T cell receptor (TCR) and can express either CD4 or CD8 molecules, allowing the identification of CD4^+^ T helper cells (Th) and CD8^+^ cytotoxic T lymphocytes (CTLs). While CTLs can exert direct cellular cytotoxicity, Th cells are required for the initiation of humoral and cell-mediated immune responses. Thus, they can be divided into several subtypes based on functions and the production of specific cytokines - Th1, Th2, Th17, Tfh and Treg cells (159). The involvement of T cells through various mechanisms in the development and progression of chronic inflammation is undisputed (160–163). T cell function in inflammation is highly pleotropic and dependent on intra- and intercellular communication, which is often mediated by N-glycans and their corresponding binding partners (**Figure 4**). In this regard, alterations in the N-glycome of T cells can significantly affect their activation, differentiation, survival, and cytokine production, often leading to autoimmunity, chronic inflammation, or cancer (164). Under homeostatic conditions, galectins are the major immune regulators of T cells, with Gal-1, Gal-3, and Gal-9 consistently showing immunosuppressive effects. The role of galectins in immunomodulation of T cells has been discussed in detail by several authors (111, 128, 129, 165). Therefore, we will specifically discuss galectin functions mediated by N-glycosylation in chronic inflammation, along with the latest findings on the underlying mechanisms affecting N-glycosylation in and by inflammation itself. Gal-1 and Gal-3 preferentially bind to branched N-glycans containing the LacNAc motif found on their T cell counter-receptors such as CD7, CD45, CD43 and TCR. This leads to inhibited transendothelial migration and induced apoptosis of T cells (108). The aforementioned binding is under the direct influence of the activity of glycan-modifying enzymes and the availability of corresponding substrates. One such enzyme is Golgi Beta-1,6-N-acetylglucosaminyltransferase V (MGAT5), which catalyzes the biosynthesis of tetra-antennary N-linked glycans, the preferred intermediates for elongation with (poly) LacNAc and ligands for galectins. MGAT5 expression in T cells is altered in chronic inflammatory diseases at both the genetic and protein levels. At the genetic level, several *MGAT5* single-nucleotide polymorphisms (SNPs), associated with reduced expression of the MGAT5 enzyme, have been found to correlate with pathological changes in T cell glycosylation in chronic diseases such as IBD, COPD, and multiple sclerosis (MS) (47, 166, 167). Deficiency in the N-glycosylation branching pathway increases susceptibility to development of severe forms of disease due to the lack of galectins’ binding substrate and consequently their inability to inhibit the exuberant Th1/Th17 immune response (168, 169). In addition, N-glycosylation alterations may occur under the influence of various cytokines; an interesting study showed that in chronic viral infection, IL-10 induced expression of MGAT5 in CD8^+^ T cells promotes the formation of the Gal-3 lattice and increases the antigen activation threshold. Normally, this would be considered an anti-inflammatory mechanism, but this restriction in viral infection allows rapid replication of the pathogen, and thus leading to the establishment of persistent chronic inflammation (170). In terms of cytokine-mediated T cell N-glycome regulation, IL-2 is one of the most involved. Based on their research in MS, Grigorian and colleagues elegantly explained the paradoxical impacts of IL-2 on N-glycan branching and MGATs in T cells. Interestingly, IL-2 reduces N-glycan branching in resting T cells, whereas it has the opposite effect in activated T cells. This is thought to be a consequence of IL-2 induced upregulation of MGAT1, an enzyme that catalyzes the biosynthesis of mono-branched N-glycans, in resting T cells. MGAT1 has a ∼250-fold higher affinity for UDP-GlcNAc than MGAT5, thus increased MGAT1 expression inhibits further N-glycan branching by limiting UDP-GlcNAc availability to MGAT5. In contrast, in active T cells, TCR signaling appears to increase levels of MGAT5 and UDP-GlcNAc, thereby exploiting IL-2 induced upregulation of MGAT1 to increase N-glycan branching by providing more substrates for downstream enzymes (47, 171). IL-2 is also involved in T cell differentiation. It suppresses the formation of Th17 and Tfh while promoting the development and activation of Treg cells (172–174). The latter is critical for maintaining immune homeostasis, as Treg cell dysfunction is associated with several inflammatory diseases. Therefore it is no surprise that low-dose IL-2 therapy has shown improvement in various autoimmune and inflammatory conditions (175–177). Also, mature Treg cells on their surface carry IL-2R receptor consisting of three subunits, IL-2Rα (CD25), IL-2Rβ (CD122), and IL-2Rγc (CD132), of which CD25 is heavily N- and O-glycosylated (178). Reduced branching decreases surface expression and retention of CD25, inhibits proper IL-2 binding, and eventually prevents Treg cell activation which consequently promotes inflammation (179). In addition to glycosyltransferase activity, substrate availability is another critical factor for successful N-glycan branching. The hexosamine biosynthetic pathway (HBP) is the main source of UDP-GlcNAc, which is required for N-glycan branching. *De novo* synthesis of UDP-GlcNAc is characterized by the conversion of fructose-6-phosphate to glucosamine-6-phosphate by the rate-limiting enzyme glutamine-fructose-6-phosphate transaminase (GFPT). To complete the conversion, GFPT also requires glutamine. Thus, the synthesis of UDP-GlcNAc by HBP may directly compete with glycolysis and glutaminolysis for fructose-6-phosphate and glutamine, respectively (179, 180). Inflammatory Th1 and Th17 undergo a metabolic switch from oxidative phosphorylation to glycolysis and glutaminolysis during inflammation (181). Therefore, by switching to glycolysis alone during inflammation, Th1/Th17 indirectly starve the hexosamine pathway of fructose-6-phosphate and consequently UDP-GlcNAc. In addition, Th17 cytokines were shown to induce down-regulation of GFPT, UDP-GlcNAc and branching in abundantly present proinflammatory T cells. These data suggest that glycolysis drives Th17 over Treg differentiation, with Th17 cytokines further maintaining reduced N-glycan branching (179). Therefore, a potential treatment for autoimmune diseases could be with metabolites of the hexosamine pathway (180). As can be seen, alteration of N-glycan branching seems to have dual function in promoting inflammation; it abrogates immunosuppression by galectins and shifts fate toward inflammatory T cells. Nevertheless, N-glycan branching is not the only feature that influences immune modulation and polarization of T cells. It has long been known that Gal-1 preferentially kills proinflammatory Th1 cells over anti-inflammatory Th2 and Treg cells. The latter is explained by the fact that Th2 and Treg cells have higher expression of ST6GAL1, which is responsible for the synthesis of terminal α2,6-sialic acids, compared with Th1 cells, and are thus protected from galectin-mediated apoptosis (109, 130, 182). Not surprisingly, the expression of ST6GAL1 is altered in chronic inflammation. In SLE, the expression of ST6GAL1 is increased in autoimmune-activated T cells, which inhibits the binding of Gal-1 and thus contributes to the pathophysiology of SLE (183). Moreover, besides the lymphocyte-specific ST6GAL1, there is a soluble form of ST6GAL1 released from the liver which is also involved in the immunomodulation of T cells. Interestingly, in mice with hepatocyte-specific ablation of ST6GAL1, there was an increase in local inflammation and a decrease in systemic Ag tolerance projected *via* increased T cell activation, and thus greater susceptibility to T cell dependent inflammatory diseases. Paradoxical as this may seem with respect to galectin inhibition, this clearly demonstrates that galectins are not sufficient to carry T cell immunosuppression alone. This is consistent with the recent discovery that liver macrophages expressing the α2,6-sialic acid-specific Siglec, CD22, can inhibit α2,6-sialic acid decorated T cells, which provides an alternative liver-driven mechanism for maintaining systemic immune homeostasis (184). APCs also have a key role in T cell polarization and activation. Sialylation of antigens has been shown to cause a shift in the differentiation of effector T cells toward tolerogenic Treg through the sialic acid-siglec axis on DCs. This could open a new way to treat patients suffering from autoimmune diseases or allergies (142, 185). Finally, another important glycosylation trait on T cells that is altered in chronic inflammation is fucosylation. The TCR receptor requires core fucosylated N-glycans for its proper activation and function. This is mediated by the Alpha-1,6-Fucosyltransferase, FUT8. In SLE and IBD, the expression of FUT8 is strongly upregulated, resulting in a hyperfucosylated TCR and thus hyperactivated T cells that contribute to the pathophysiology of the aforementioned diseases (186, 187). On the other hand, core fucosylation is required for the expression of programmed cell death receptor 1 (PD-1), which is responsible for attenuating TCR signaling, resulting in depleted and unresponsive T cells (188). The hyper-core fucosylation induced upregulation of PD-1 expression could then provide an explanation for the impairment of T cells in chronic viral infections (189, 190). Unfortunately, the underlying mechanism of upregulated core fucosylation in chronic inflammation is still unclear and is a topic for further study. In summary, inflammation has apparently found every loophole in the N-glycosylation life cycle of T cells to turn the tide in its favor. Therefore, it is necessary to consider N-glycosylation during the development of anti-inflammatory therapy, and particularly in case of a therapy specifically targeting critical steps in the transition from homeostasis to inflammation.

### B Cells

**Figure 5:**
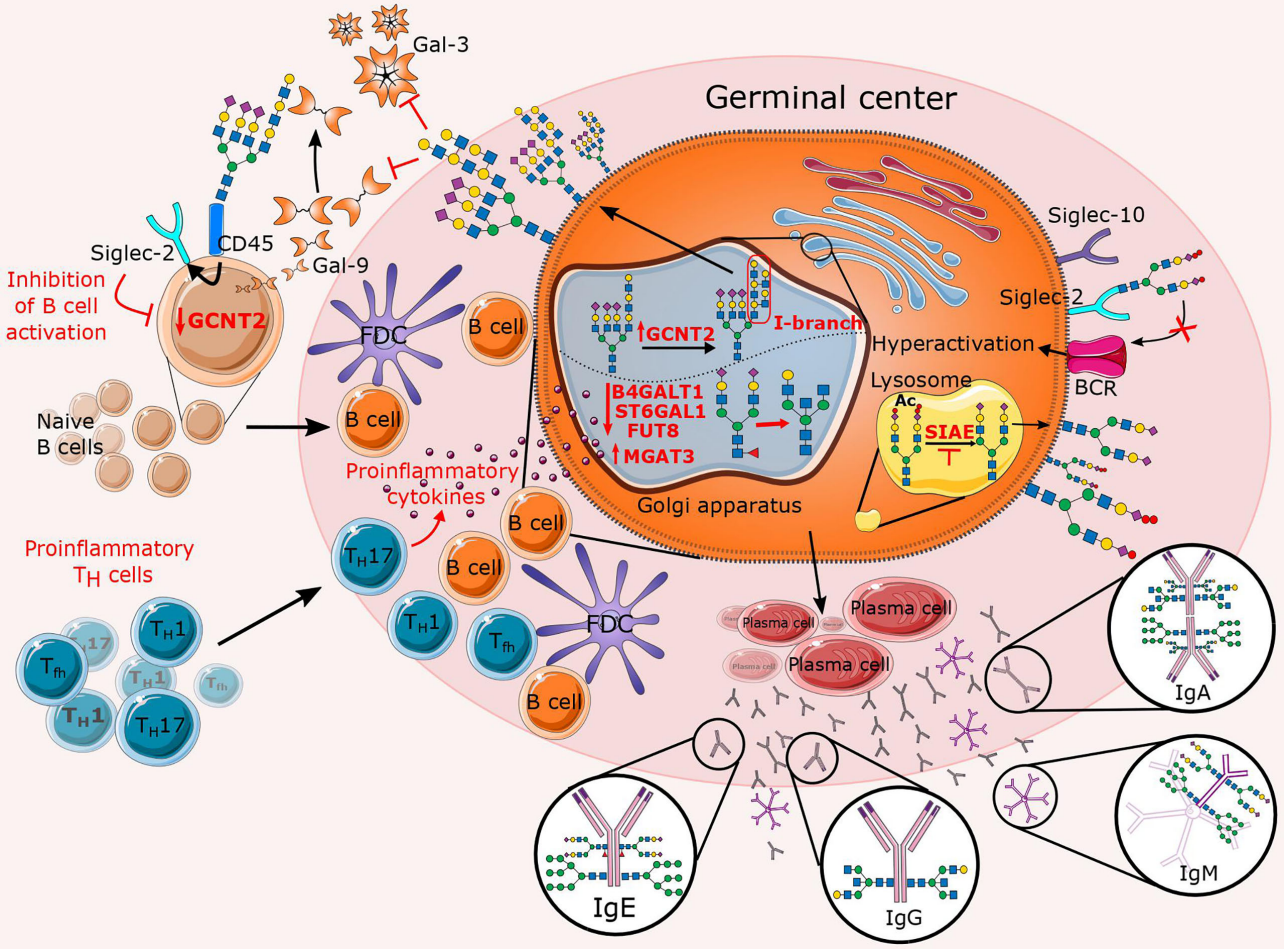
Overview of altered N-glycosylation pathways regarding B cells during chronic inflammation. In the presence of proinflammatory stimuli, inflammatory T cells significantly affect B cell proliferation and their N-glycan profile by deregulating a specific subset of glycosyltransferases (B4GALT1, ST6GAL1, FUT8, MGAT3, and GCNT2). The latter is reflected in an increase in features such as bisecting GlcNAc, agalactosylation, afucosylation, and the presence of I-branches that have been shown to inhibit Gal-3 and Gal-9 binding. In addition to the affected Golgi enzymes, lysosomal sialic acid acetyl esterase (SIAE) is also downregulated so that it is unable to deacetylate sialic acids, which is necessary for immunomodulation of B cell receptor (BCR) signaling. This figure also summarizes the Fc N-glycome of secreted immunoglobulins, which reflects inflammation-related changes that may further contribute to disease progression.

B cells, also called B lymphocytes, are the major central effector immune cells in the humoral branch of adaptive immunity. During inflammation, naïve or memory B cells are exposed to antigens by APCs under co-stimulation of Th cells in the germinal center (GC) in secondary lymphoid organs. This induces activation and rapid proliferation of B cells and selection of high-affinity B cell receptors (BCRs) (191, 192). B cells expressing a high-affinity receptor enter the periphery, where they differentiate into plasma cells that secrete large amounts of antibodies (193). Once antibodies encounter their antigen, pathogen, or infected cells, their functions include neutralization, ADCC, phagocytosis, and complement-dependent cytotoxicity (CDC) (194). In addition to their function as precursors of antibody-secreting plasma cells, B cells are involved in suppression of T cells and secretion of relevant cytokines that control adaptive immunity (195, 196). N-glycosylation has a tremendous impact on B cell proliferation, differentiation, and effector functions (**Figure 5**), but research on this topic lags far behind that of T cells. Nevertheless, there are implications that altered N-glycosylation in B cells may contribute to the development of various chronic inflammatory (autoimmune) diseases.

In B cells, among the best understood roles for lectin-glycan interactions are those of sialoglycans and Siglecs in BCR signaling. Sialic acids are often referred to as inhibitory “self-signals” because of their high local concentration on the surface of B cells. Thus, it is not surprising that Siglecs are considered major B cell immunomodulators (197). B cells express siglec-2 (CD22) and siglec-10 (ortholog of mouse siglec-G), both of which preferentially bind α2,6-sialic acid and act as inhibitory co-receptors of the BCR to maintain peripheral tolerance and prevent autoimmunity (198, 199). Thus, varying degrees of autoimmunity have been observed in mice lacking CD22, Siglec G, or both (200–202). Interestingly, ST6GAL1 deficient B cells show suppressed BCR signaling, yet mice deficient in both CD22 and ST6GAL1 showed restored BCR signaling, emphasizing the importance of α2,6-sialic/siglec axis in immunomodulation of BCR signaling (203). In addition to ST6GAL1, sialic acid acetyl esterase (SIAE) is another enzyme involved in regulation of BCR signaling. SIAE deacetylate sialic acid ligands, thereby allowing CD22-mediated inhibition of BCR signaling (204). Several studies showed that congenital mutations in SIAE are associated with an increased risk of autoimmune disease in humans (205–207). Nevertheless, while the contribution of sialic acid/siglec interactions to B cell function has been extensively defined (97, 199, 208, 209), sialoglycans represent only a fraction of the glycans involved in interactions regulating B cell immunity. Recently, Giovannone and colleagues discovered that B cells express significant amounts of tri- and tetra- complex N-glycans decorated with (poly)LacNAcs. Interestingly, the (poly)LacNAc structures were linear on naïve B cells but modified by Glucosaminyl (N-acetyl) Transferase 2 (GCNT2) with I-branches in GC B cells. In addition, I-branches were found to selectively impair B cell binding to Gal-9. Specifically, Gal-9 was found to be predominantly expressed by naïve B cells and to bind mainly the glycoprotein receptor CD45 carrying (poly)LacNAc decorated N-glycans. Functionally, binding of Gal-9 to CD45 induces inhibitory signaling through interaction with CD22, abrogated BCR calcium flux, and attenuated B cell activation. On the contrary, in GC B cells, Gal-9 mediated inhibition is down-modulated by the combined downregulation of Gal-9 and upregulation of GCNT2 (210). Although this is a novel BCR regulatory axis involving Gal-9 and GCNT2, further studies are needed to unravel the underlying mechanisms controlling the expression of these proteins. However, increased expression of Gal-9 has been reported in various autoimmune inflammatory diseases (211), therefore the exact involvement of Gal-9 in B-cell (dys)function in chronic diseases remains to be elucidated. In addition to Gal-9, other galectins such as Gal-1 and Gal-3 are also known to regulate BCR signaling, plasma cell differentiation, and survival (212–214). Interestingly, besides Gal-9, I-branches have also been shown to selectively impair B cell binding to Gal-3 but not Gal-1 (210, 215). A proposed explanation includes the fact that Gal-3 and Gal-9 preferentially bind to internal LacNAc residues, while Gal-1 favors binding to (poly)LacNAc termini (128). This suggests that I-branches may inhibit Gal-3 and Gal-9 binding, whereas terminal modifications such as α2,6-sialylation by ST6GAL1 may more selectively inhibit Gal-1. In addition, branched N-glycans are not exclusively a feature of B cell regulation but also of B cell mediated T cell regulation in autoimmunity. Branched N-glycans have been reported to suppress B cell triggered proinflammatory Th1/Th17 differentiation by promoting Toll-like receptor-2 (TLR2) and Toll-like receptor-4 (TLR4) endocytosis and downstream APC activity in B cells, thereby reducing inflammatory demyelination in a murine model of MS. At the same time, it was observed that minimal branching promotes surface retention of BCR and its co-receptor CD19, thereby stimulating adaptive B cell function. Although branching may represent another way to prevent Gal-9 binding and enhance BCR signaling, MGAT1 deficiency has been observed to prevent both branching and poly-LacNAc synthesis, leading to a decrease in BCR/CD19 surface expression and BCR signaling in this MS model (216). Because altered expression of glycosyltransferases may be a mechanism of differential regulation of galectin activity and receptor expression in B cells, future studies are needed to determine potential roles of these enzymes and their corresponding effector molecules in the development of autoimmune diseases. Lastly, it was demonstrated that core fucosylation of IgG-BCR mediates antigen recognition, along with cell signal transduction *via* BCR and antibody production (217).

The final, but not less important, role of B cells is the secretion of immunoglobulins (Igs) - the major executive glycoproteins of the humoral adaptive immune response. In humans, five classes of immunoglobulins exist: IgG, IgA, IgE, IgM, and IgD. All human Ig classes are N-glycosylated, with N-glycans attached to the conserved glycosylation regions on the fragment crystallizable (Fc) and/or on the variable fragment antigen binding (Fab), where new glycosylation sites can be acquired during somatic hypermutation (218). N-glycans can affect the structural stability and conformation of immunoglobulins as well as their effector functions (219). While alterations in N-glycosylation of IgG have been observed in several chronic (inflammatory) diseases and discussed in detail elsewhere (3), not much is known about alterations in the N-glycosylation profile of other immunoglobulins under pathological conditions. In the following paragraphs, the current knowledge about this topic is summarized with the focus on the possible underlying mechanisms mediated by inflammation that could contribute to the alterations in N-glycosylation of Igs.

#### IgG

IgG represents 75% of all antibodies in human serum, making it the most abundant immunoglobulin class in the bloodstream (220). Its Fab region recognizes and binds antigens, while the Fc fragment interacts with type I and type II Fcγ receptors (FcγRs) on the surface of many immune cells (including macrophages, neutrophils, B cells, NK cells, etc.), triggering various immune responses such as antigen neutralization, macrophage phagocytosis, ADCC, and complement activation (221). Each IgG molecule contains a conserved N-glycosylation site at the Asn297 of the constant heavy 2 (CH2) domain on each of its heavy chains. This site is where most of the contact with the various IgG Fc receptors and ligands occurs, and it is critical for maintaining both the pro- and anti-inflammatory effector functions of IgG (222). Glycosylation traits that are of most importance for IgG effector functions, and so mostly altered in/by inflammation, are galactosylation, sialylation, fucosylation and bisecting GlcNAc.

##### Galactosylation

Increased abundance of agalactosylated IgG glycans is considered a hallmark of various diseases with an underlying inflammatory component (3). Fc glycans lacking terminal galactoses are thought to be proinflammatory by activating complement through the alternative pathway along with the lectin pathway by binding to mannose-binding lectin (MBL) (223, 224). While agalactosylated glycans are considered strictly proinflammatory, terminal galactosylation seems to be quite controversial in this regard. Glycans decorated with galactoses have been held responsible for attenuating inflammation by binding to the inhibitory FcγRIIB, followed by inhibition of the proinflammatory activity of complement component C5a (225). On the other hand, Fc galactosylation is shown to activate the classical complement pathway by facilitating IgG hexamerization, thereby increasing C1q avidity and enhancing CDC (226). It has also been found to increase the affinity of IgG for activating FcγRs, leading to ADCC (227, 228). Although biological functions of (a)galactosylated IgGs are described, the underlying mechanism of how this is regulated in inflammation remains unclear. In this context, decreased levels of IgG galactosylation have been shown to associate with decreased activity of Beta-1,4-Galactosyltransferase 1 (B4GALT1) in peripheral B cells from RA patients, but no difference in expression of B4GALT1 was observed in RA patients compared with healthy controls. Proposed explanation points toward a stress-induced disruption of Golgi (heat shock and other stress proteins are elevated in RA), which could affect the proper targeting of B4GALT1 and thus impair its catalytic function (229). On the other hand, proinflammatory cytokines are observed to alter glycosylation of IgG indirectly *via* T cell-dependent (TD) activation of B cells. Accordingly, low levels of IgG galactosylation were dependent on the effects of the Th1 cytokine interferon γ (IFN-γ) *via* IFN-γRI signaling, as decreased agalactosylation was observed in Ifngr1^-/-^ mice (230). To support this, a novel B-cell intrinsic IFN-γR signaling pathway has been defined that is required for Tfh cell development and promotes autoreactive B cell formation and autoimmunity (231). Tfh cells secrete cytokines such as interleukin 6 (IL-6), IFN-γ, and interleukin 17 (IL-17), which maintain the agalactosylated state of IgGs (232). Also, a recent genome-wide association study (GWAS) showed that IL-6 signaling [SNPs in the *IL6ST* (gp130) gene] correlates with low serum IgG galactosylation (233). Interestingly, binding of these cytokines to their receptors leads to activation of JAK/STAT pathway known to target genes that appear to promote inflammation (234), therefore it is plausible that targeted genes include galactosyltransferases.

##### Sialylation

The addition of sialic acid to the terminal end of IgG N-glycans is essential for the control of inflammatory immune responses. Highly sialylated IgG have a lower affinity for activating FcγRIIIa, resulting in reduced ADCC (235, 236), whereas they stimulate upregulation of inhibitory FcγRIIb and thus inhibition of CDC (237). In autoimmunity, hyposialylation is thought to be responsible for the development of chronic inflammation. The results of more in-depth studies have shown that IL-23 stimulates Th17 cells to secrete IL-21 and IL-22, which are responsible for decreased expression of ST6GAL1, and thus sustaining hyposialylated state of IgG (238). Another explanation for IgG hyposialylation includes Tfh cells, and especially Tfh17 and Tfh1 cells. Tfh17 cells negatively regulate ST6GAL1 from autoantibody-producing B cells *via* the OX40-OX40L (TNF receptor superfamily) interaction. An increased number of OX40-overexpressing Tfh17 cells was observed in RA patients, and their frequency was negatively correlated with ST6GAL1 expression. However, blocking the OX40-OX40L pathway resulted in a decrease of Tfh17 cells and upregulation of IgG sialylation (135). Moreover, IL-27 stimulates Tfh1 to secrete IFN-γ, which can downregulate ST6GAL1 expression in cultured B cells by binding to the B cell intrinsic IFN-γR, and activating the JAK1/2 signaling pathway (232). Consistent with this effect of T cell cytokines on sialylation of IgG, it has been shown that T cell-independent B cell activation leads to the development of immunosuppressive sialylated IgG capable of abrogating B cell activation independent of FcγRIIb (230), possibly promoting an inhibitory feedback mechanism by binding to CD22 expressed on the B cell surface (239). In addition to inflammatory cytokines, increased risk of RA under conditions of low estrogen levels (e.g., menopause) correlate with estrogen induced increase in IgG Fc sialylation through increased expression of ST6GAL1 in splenic plasmablasts (240). Of note, recent evidence suggests that IgG glycans can be extracellularly sialylated by hepatic ST6GAL1 present in the bloodstream (241, 242), although this appears to be an inflammation-dependent process rather than a constitutive one (243).

##### Core Fucosylation

More than 90% of Fc glycans of IgG in healthy individuals have fucose bound to their core, which acts as a “safety switch” and attenuates potentially harmful ADCC (94). More recently, decreased fucosylation of the IgG core has been found in autoimmune thyroid diseases. The underlying mechanism is thought to be abnormal expression of the *FUT8* and *IKZF1* genes in B cells producing thyroid peroxidase antibody (TPOAb) (244). Both genes have previously been associated with afucosylated IgG N-glycans (233). Although the exact mechanism is still unclear, the *IKZF1* gene encodes the transcription factor Ikaros, a potential indirect regulator of fucosylation in B cells by promoting the addition of bisecting GlcNAc, which then inhibits fucosylation (233). Interestingly, several SNPs surrounding the *IKZF1* gene have been associated with other autoimmune diseases, including SLE (243) and IBD (244). Of note, elevated plasma levels of α-L-fucosidase (FUCA-1) were significantly associated with chronic inflammation and autoimmune diseases (245), raising the question of extracellular IgG defucosylation in inflammation. On the contrary, Plomp et al. found that IgG fucosylation is increased in individuals with a higher degree of inflammation, sometimes even in autoimmune patients (246). This was further investigated by Huang et al. and they found that increased IgG core fucosylation was observed in the serum of RA patients with a concomitant decrease in α2,6-sialylation. Moreover, α2,6-sialylation of IgG was increased in Fut8^-/-^ mice (247). These findings may represent a novel mechanism for disease-specific, inflammation-related changes in IgG glycome that are consistent with distinctive observations regarding fucosylation and sialylation in autoimmune diseases differing in mechanisms of pathophysiology.

##### Bisecting N-Acetylglucosamine (GlcNAc)

Bisecting GlcNAc has been classified as a proinflammatory trait in many inflammatory diseases (3). Although afucosylated IgG plays the most important role in enhancing ADCC, the addition of bisecting GlcNAc to IgG Fc glycans has also been reported to boost ADCC (248). However, because the presence of bisecting GlcNAc blocks the addition of the core fucose residue (233, 249), it is difficult to distinguish the functional roles of these two glycosylation features (248). Nevertheless, epigenetic modifications and proinflammatory stimuli are shown to be responsible for increased abundance of bisecting GlcNAc on IgG Fc glycans in inflammation. It has been demonstrated that aberrant methylation in the promoter region of the *MGAT3* gene (encoding the MGAT3 enzyme responsible for the production of bisecting GlcNAc structures) results in an increased percentage of bisecting GlcNAc on IgG glycans in CD patients, suggesting a possible involvement of bisecting GlcNAc in the pathogenesis of CD (168). Moreover, Ho et al. demonstrated that the cytokine transforming growth factor β1 (TGF-β1) exerts paradoxical activity, depending on the inflammation state, in relation to the presence of tissue fibrosis and bisected IgG (250). Although further studies are needed to derive specific mechanisms that influence the formation of bisected IgG, the functional importance of this feature in inflammation is undisputed.

#### IgA

Immunoglobulin A (IgA) is by far the most abundant antibody in the human body (251). The majority of IgA is secreted as a dimer and is known for its protective role on mucous membranes. In serum, IgA is the second most abundant isotype, usually produced as a monomer (252). For a long time, IgA was considered ‘passive’ or anti-inflammatory, but recently it has become clear that IgA also actively triggers immune responses. IgA can trigger inflammation *via* FcαRI (CD89) by directing the secretion of cytokines. Therefore, its involvement in the pathogenesis of various chronic inflammatory diseases (253) is not surprising. IgA has two conserved N-linked glycosylation Fc sites (Asn263 and Asn459) (254), but there are limited data on how the Fc N-glycome of IgA modulates binding to FcαRI. The fact that FcαRI has no direct mouse homolog (255, 256) may be a possible explanation for the lack of research on this topic. Nonetheless, N-glycosylation of IgA appears to be associated with inflammation. One of the best studied chronic inflammatory diseases related to IgA N-glycosylation is IgA nephropathy (IgAN). Recently, a study by Dotz et al. showed that a decrease in N-linked sialylation and galactosylation, and increased bisection in IgAN is associated with worsening renal function (257). Interestingly, it has been shown that mice lacking B4GALT1 develop human IgAN-like glomerular lesions and have high serum levels of polymeric IgA with agalactosylated N-glycans (258). The elevated levels of polymeric form of IgA in patients with IgAN is also associated with increased immune complex formation (259). While monomeric IgA induces inhibitory immunoreceptor tyrosine-based activation motif (ITAMi) signaling *via* FcαRI, binding of IgA immune complexes to FcαRI triggers classical ITAM signaling and activates inflammatory responses (260, 261). Furthermore, quantitative analysis revealed significant differences in N-linked glycosylation between monomeric IgA and polymeric IgA, including the presence of oligomannose exclusively on polymeric IgA (262). The differential N-glycosylation of polymeric IgA may contribute to its enhanced binding to mesangial cells and their subsequent activation, as well as to its ability to activate complement *via* binding to MBL. Moreover, the absence of terminal α2,6 linked sialic acid enhances the pro-inflammatory capabilities of IgA (263) and may serve as a predictor of poor prognosis in patients with IgAN (264). On the contrary, elevated plasma ST6GAL1 levels have been shown to be associated with IgAN disease severity (265), possibly representing an anti-inflammatory positive feedback loop. Overall, these findings may suggest a link between N-glycosylation of IgA and the pathogenesis of IgAN *via* increased formation of polymeric IgA. However, further in-depth studies are required for a better understanding of the potential role of IgA N-glycome in the development and progression of inflammatory diseases.

#### IgE

Immunoglobulin E (IgE) is best known for its role in allergic immune responses. Specifically, IgE binds to high-affinity IgE receptors (FcϵRI) expressed on the surface of basophils and mast cells, triggering degranulation and the release of proinflammatory mediators (266). IgE is the most glycosylated immunoglobulin, having seven N-glycosylation sites (267). However, because IgE is the least abundant immunoglobulin in the bloodstream (268), analysis of N-glycosylation of IgE is significantly limited, leaving the biological function of IgE N-glycosylation largely unclear. However, it has been shown that there is a single N-glycosylation site at Asn394 consisting exclusively of oligomannose N-glycans which is critical for IgE-mediated initiation of the allergic cascade. Specific amino acid mutations or complete deglycosylation of Asn394 alter the secondary IgE structure, abolishing FcϵRI binding and subsequent IgE-mediated degranulation and anaphylaxis (269, 270). Interestingly, mutation of all other N-linked sites of IgE, which consist of complex N-glycans, had almost no effect on the ability of IgE to elicit an anaphylactic response (270). Although the underlying mechanism is not yet known, the functional significance of oligomannose N-glycans at Asn394 may provide a unique therapeutic target. On the other hand, galectins such as Gal-3 and Gal-9 have also been shown to be involved in the regulation of IgE-mediated functions. Gal-3, previously known as IgE-binding protein, has the ability to cross-link IgE and FcϵRI *via* their N-glycans and trigger basophil or mast cell activation (271). Moreover, both Gal-3 (272) and IgE (273) are overexpressed in atopic dermatitis (AD), suggesting that they are important players in mediating chronic inflammation in AD. In contrast, Gal-9 has been shown to reduce mast cell degranulation and anaphylaxis by blocking the formation of the IgE-antigen complex (274). Given the affinity of these galectins for complex N-glycans (128), it is likely that the galectin-IgE interactions mentioned above are mediated by complex N-glycans on IgE. Strikingly, the removal of terminal sialic acid on IgE N-glycans, as well as coexistence of other asialylated glycoproteins, attenuates degranulation of effector cells (275). The exposed terminal galactoses could exert a suppressive function by binding to inhibitory galectins, although the exact mechanism remains to be elucidated.

#### IgM

Immunoglobulin M (IgM) is the largest antibody in serum and its level is elevated in various inflammatory and autoimmune diseases (276). It is another highly N-glycosylated antibody, as its constant domain contains five N-linked glycosylation sites, three of which belong to the biantennary complex form (Asn171, Asn332, Asn395) and two to the oligomannose type (at Asn402, Asn563) (219). Oligomannose N-glycans have been shown to be important for MBL binding and subsequent elimination of IgM aggregates by opsonization (277). On the other hand, complex N-glycans are involved in immunomodulation of T and B cells. Sialylated N-linked glycans have been demonstrated to induce internalization of IgM by T cells, which in turn causes inhibition of T cell responses. The authors hypothesized that IgM-mediated immunosuppression occurs through the binding of sialylated IgM to the constitutively expressed IgM Fc receptor (FcμR) on the surface of T cells (278). On the other hand, B cell activation is under the direct influence of Gal-9-mediated negative regulation. It has been proposed that Gal-9 organizes IgM-BCR and the inhibitory molecules CD45 and CD22 into larger clusters by binding to their N-linked glycans, and thus directly inhibiting BCR signaling (279). Considering Gal-9 binding preferences (128), the above N-glycan-mediated interaction could be facilitated by complex N-glycans on IgM molecules. In addition, sialylated N-glycans on soluble IgM are preferential *trans*-binding ligands for CD22, which further contributes to the abrogation of BCR signaling (280). These results support the concept that the presence of α2,6-sialic acid on Igs contributes to immunosuppression, as previously demonstrated for the anti-inflammatory effects of intravenous immunoglobulin therapy (IVIg) (281).

#### IgD

Even though O-glycans of Immunoglobulin D (IgD) are associated with autoimmune diseases (282), nothing is known about the role of N-glycosylation in IgD effector functions, despite having three N-glycosylation sites in the Fc domain (Asn354, Asn445, Asn496) (283). The oligomannose glycans at Asn354 are inaccessible for potential lectin interactions because the complex N-glycans at Asn445 block binding (284). Nevertheless, oligomannose N-glycans are critical for IgD production, and elimination of the Asn354 site by mutagenesis results in incomplete assembly and failure of secretion (285), proposing that the N-glycans are necessary for maintenance of the correct Fc structure, which is important for IgD secretion.

## Acute Phase Proteins

APPs are mainly synthesized and secreted by hepatocytes. During inflammation, proinflammatory cytokines such as IL-1, IL-8, IL-6, and TNFα stimulate the acute phase response (286–289), increasing APP serum levels up to 1000-fold (288). Several APPs are glycoproteins and changes in their N-glycans have been observed in chronic inflammation. The most significant N-glycosylation changes observed in APPs are high branching (tri- and tetra-antennary glycans) and increased levels of sLex epitope as detected on haptoglobin (HPT), α1-acid glycoprotein (AGP-1), α1-antitrypsin (A1AT), and α1-antichymotrypsin (ACT) (29, 290–292). The sLex epitope on AGP contributes to its antineutrophil capacity (75) and is critical for binding to endothelium-expressing E-selectin, where AGP competes with sLex-expressing leukocytes, providing a feedback inhibition mechanism (293). Proinflammatory cytokines IL-1β, IL-6, and TNFα, involved in the induction of the acute phase response, may also be involved in the regulation of APP glycan biosynthesis in hepatocytes (294–298). *In vitro* studies have shown that AGP expresses N-linked glycans with increased branching and sLex epitope when hepatocytes are stimulated with IL-1β and IL-6 (294), possibly through cytokine mediated upregulation of enzymes responsible for biosynthesis of sLex epitope, ST3GAL4 and FUT6 (295). Furthermore, TNFα has also been shown to increase sLex synthesis by stimulating the expression of ST3GAL4 and FUT4 *via* NFkB-p65 dependent transcriptional regulation (298, 299). In addition to *in vitro* studies, TNFα induced increase in sLex epitope has also been observed in RA patients (300). Based on the results of their GWAS study, Lauc et al. described another pathway for the regulation of plasma protein sLex formation involving hepatocyte nuclear factor 1α (HNF1α) and its transcriptional cofactor HNF4α. HNF1α/HNF4α induce both *de novo* and salvage synthesis of GDP-fucose, upregulate antennary fucosyltransferases (FUT3/4/6) and downregulate core fucosyltransferase (FUT8), ultimately leading to increased sLex-expressing APPs (301). Interestingly, HNF1α mediated transactivation of hepatic genes is stimulated by IL-6 (302), adding to the molecular mechanism behind the reported association between proinflammatory cytokines and increased levels of sLex-expressing APPs. While sLex epitope formation is highly dependent on cytokine mediated increase in the expression of relevant glycosyltransferases, increased HBP flux and consequently higher levels of UDP-GlcNAc in hepatocytes lead to increases in tri- and tetra-antennary N-glycans on APPs in chronic inflammation. Donor molecules directly involved in modulating UDP-GlcNAc levels and HBP flux are glucose and glutamine (303). During sustained inflammation, increased hepatic uptake of glutamine and increased hepatic glucose production *via* TNFα-activated NF-kB transcriptional regulation have been observed (304, 305). Consequently, increased hepatic HBP flux leads to high levels of UDP-GlcNAc, the crucial substrate for N-glycan multistep branching of APPs and other hepatic glycoproteins. The biosynthesis of tri- and tetra-antennary N-glycan-decorated APPs is ultrasensitive to UDP-GlcNAc content, as the affinity for UDP-GlcNAc decreases from MGAT1 to MGAT5 (306). Furthermore, N-glycan branching of hepatic membrane transporters (for glucose and glutamine) increases galectin binding affinity, protecting them from endocytosis and thus establishing a positive feedback loop by increasing HBP substrate uptake (307). In addition to the aforementioned APPs, hepatic ST6GAL1 is also upregulated and released into the circulation during inflammation (308). Although certain anti-inflammatory effects of hepatic ST6GAL1 have been observed (242, 309), its role still remains elusive. However, Oswald and coworkers have shown that loss of hepatic ST6GAL1 leads to dysregulation of hepatic metabolic pathways and consequent changes in the N-glycan profile of circulating glycoproteins. It has been observed that loss of α2,6-sialic acid, core and/or antennary fucose, and an increase in α2,3-sialylation, branching, and bisection ultimately lead to spontaneous liver inflammation and disease (310). Interestingly, chronic alcohol exposure has previously been shown to downregulate hepatic *ST6GAL1* gene expression, leading to metabolic dysfunctions, including altered glycosylation (311). This highlights the fact that lifestyle may contribute to the loss of hepatic ST6GAL1, which in turn triggers the development of inflammation and activates the cascade of proinflammatory cytokines responsible for the increased expression of hepatic ST6GAL1 during the peak of inflammation (308, 309, 312, 313), providing a positive feedback loop that may explain hepatic ST6GAL1 paradox.

## Conclusion

N-glycosylation is one of the key mediators in intercellular interaction and communication, which makes it highly susceptible to changes in inflammation. On the other hand, as discussed above, altered N-glycosylation affects the immune response, which may further enhance the inflammatory reaction. Therefore, N-glycans are essential for normal immune system function, from innate to adaptive immunity. This opens up the possibility for development of new therapeutic approaches for various inflammatory diseases targeting altered N-glycan structures or biosynthetic enzymes associated with glycosylation. Moreover, the potential of N-glycosylation alterations as novel biomarkers or as enhancements of existing ones for disease predisposition and progression, as well as for diagnosis, prognosis, and response to therapy, cannot be ignored. However, further in-depth research is needed to elucidate the precise mechanism underlying some of these alterations so that these discoveries can be translated into clinical practice and diagnostic test development.

## Author Contributions

BR collected the data and wrote the manuscript. IG provided valuable guidance and revised the manuscript. All authors contributed to the article and approved the submitted version.

## Funding

This research was supported by the project “GLYCARD: Glycosylation in Cardiovascular Diseases” (UIP-2019-04-5692), funded by the Croatian Science Foundation.

## Conflict of Interest

Author IG was employed by Genos Glycoscience.

The remaining author declares that the research was conducted in the absence of any commercial or financial relationships that could be construed as a potential conflict of interest.

## Publisher’s Note

All claims expressed in this article are solely those of the authors and do not necessarily represent those of their affiliated organizations, or those of the publisher, the editors and the reviewers. Any product that may be evaluated in this article, or claim that may be made by its manufacturer, is not guaranteed or endorsed by the publisher.
